# High-Field MRI Reveals a Drastic Increase of Hypoxia-Induced Microhemorrhages upon Tissue Reoxygenation in the Mouse Brain with Strong Predominance in the Olfactory Bulb

**DOI:** 10.1371/journal.pone.0148441

**Published:** 2016-02-10

**Authors:** Angelika Hoffmann, Reiner Kunze, Xavier Helluy, David Milford, Sabine Heiland, Martin Bendszus, Mirko Pham, Hugo H. Marti

**Affiliations:** 1 Department of Neuroradiology, Heidelberg University Hospital, 69120, Heidelberg, Germany; 2 Institute of Physiology and Pathophysiology, University of Heidelberg, 69120, Heidelberg, Germany; 3 Division of Experimental Radiology, Department of Neuroradiology, Heidelberg University Hospital, 69120, Heidelberg, Germany; Instituto Cajal-CSIC, SPAIN

## Abstract

Human pathophysiology of high altitude hypoxic brain injury is not well understood and research on the underlying mechanisms is hampered by the lack of well-characterized animal models. In this study, we explored the evolution of brain injury by magnetic resonance imaging (MRI) and histological methods in mice exposed to normobaric hypoxia at 8% oxygen for 48 hours followed by rapid reoxygenation and incubation for further 24 h under normoxic conditions. T2*-, diffusion-weighted and T2-relaxometry MRI was performed before exposure, immediately after 48 hours of hypoxia and 24 hours after reoxygenation. Cerebral microhemorrhages, previously described in humans suffering from severe high altitude cerebral edema, were also detected in mice upon hypoxia-reoxygenation with a strong region-specific clustering in the olfactory bulb, and to a lesser extent, in the basal ganglia and cerebral white matter. The number of microhemorrhages determined immediately after hypoxia was low, but strongly increased 24 hours upon onset of reoxygenation. Histologically verified microhemorrhages were exclusively located around cerebral microvessels with disrupted interendothelial tight junction protein ZO-1. In contrast, quantitative T2 and apparent-diffusion-coefficient values immediately after hypoxia and after 24 hours of reoxygenation did not show any region-specific alteration, consistent with subtle multifocal but not with regional or global brain edema.

## Introduction

High altitude related illness including acute mountain sickness (AMS), high altitude pulmonary edema (HAPE) and high altitude cerebral edema (HACE) can occur in non-acclimatized individuals exposed rapidly to high altitudes [1–3]. AMS is characterized by headache, anorexia, nausea, dizziness, and insomnia. HACE is considered to be the end-stage of AMS, and is accompanied by altered mental status, ataxia and progressive neurological deterioration that may ultimately result in death [1, 2, 4]. The pathophysiological mechanisms leading to HACE are poorly understood. At present, the current consensus is that HACE is caused by vasogenic edema resulting from a disruption of the blood-brain barrier (BBB). One possible explanation for vasogenic edema is that cerebrovascular autoregulation is disturbed by hypoxia leading to cerebral capillary hypertension [5]. Alternatively, cerebral capillary hypertension might be caused by impairment of cerebral venous return, especially during hypoxia-induced brain vasodilatation [6]. In addition to hemodynamic changes, biochemical mediators of BBB permeability such as vascular endothelial growth factor (VEGF) or reactive oxygen species (ROS) released during tissue hypoxia may contribute to the development of HACE [5, 7–10].

Furthermore, it is unclear if cerebral edema is of global nature or if certain predilection sites exist. So far, the splenium of the corpus callosum has been identified as predilection site in humans, as magnetic resonance imaging (MRI) in HACE survivors showed reversible T2-weighted hyperintensities in white matter areas, especially in the splenium of the corpus callosum [4]. However, further studies, simulating AMS, reported only small effects of cytotoxic edema (decrease in apparent diffusion coefficient (ADC)), a discrete combined vasogenic and cytotoxic edema (decrease in ADC and increase in T2) [1, 11, 12] or no detectable brain edema [13, 14]. These mild changes or non-detectable signs of cellular swelling or water increase are not considered to explain clinical symptoms of AMS. The unavailability of a MR scanner at high altitudes and the remission of symptoms after descent, render ‘live’ studies of AMS and HACE in humans difficult. In contrast to AMS, HACE leaves a footprint in the brain, showing microhemorrhages in the splenium of the corpus callosum and the periventricular white matter that probably resulted from blood vessel rupture [2, 15]. At present, it is also unclear whether rapid tissue reoxygenation in human mountaineers that occurs by administration of supplemental oxygen at high altitude or swift emergency helicopter evacuation to low altitude may worsen HACE and enhance the formation of brain microhemorrhages.

Thus, in the present study we used high-resolution MRI at 9.4T to study *in vivo* the temporal and spatial development of cerebral edema and microhemorrhages, as final imprints of structural brain injury, in an experimental mouse model of hypoxia-reoxygenation that might partially reflect the pathogenesis of HACE in humans.

## Material and Methods

### Animals

All experiments were performed using male adult C57BL/6 mice (10 to 14 weeks old). Mice were kept at the animal facility of the University of Heidelberg under a controlled 12:12 hours light-dark cycle, constant room temperature (22±2°C) with food and water *ad libitum*. All animal experiments were performed according to FELASA category B and GV-SOLAS standard guidelines and approved by German authorities (Regierungspräsidium Karlsruhe, Germany, §8 Abs. 1 Tierschutzgesetz (TierSchG), Approval number: 35–9185.81/G-103/12). Mice were separately placed into transparent plastic chambers (13 x 26 x 22 cm) attached to a Digamix 5SA 18/3a pump (H. Wösthoff, Bochum, Germany). Boxes have been flooded at 12 l/h with a premixed gas mixture containing room air and nitrogen in a ratio of 40:60. The initial oxygen concentration of roughly 21% (altitude: 110 m above sea level) continuously declined at 0.1% O_2_/min (corresponds to an ascent rate of 60 m/min) to 8% oxygen (corresponding to 7100 m above sea level). Mice were kept for 48 h at normobaric hypoxia. Then, rapid reoxygenation was induced by opening the chamber, and mice were maintained for further 24 h at room air (~21% oxygen). During hypoxia and after reoxygenation mice had free access to food, water and nesting material (e.g. soft paper) used as environmental enrichment.

### Magnetic resonance imaging protocol

MRI was performed on a 9.4T small animal scanner (BioSpec 94/20 USR, Bruker, Ettlingen, Germany) using a volume resonator for RF transmission and a 4-channel-phased-array surface receiver coil. An isoflurane evaporator connected to a supply of compressed air was used for anesthesia. Anesthesia was induced at 2% isoflurane and maintained with 1–1.5%. Animals were placed in prone and fixed positions on an animal holder equipped with a headlock and tooth bar to minimize head motion. Body temperature was maintained using a temperature controlled water bath. Respiration was monitored externally with an in-house developed program in LabView (National Instruments Corporation, Austin, Texas, USA).

Three MRI examinations were performed per animal: 1) baseline scan at 3 days before exposure to hypoxia, 2) immediately (within 30 min) after hypoxia and 3) at 24 hours after onset of reoxygenation. High resolution flow compensated T2*-weighted imaging was performed with a 3D RF-spoiled gradient-echo pulse sequence, as described previously by Park and colleagues [16] covering the whole brain at an isotropic resolution of 80 μm (field-of-view = 32x15x8 mm^3^, matrix size = 400x188x100, time of repetition (TR) = 50 ms, echo time (TE) = 18 ms, averages = 1, flip angle (FA) = 12°, acquisition time = 15 min 40 s). Additionally, diffusion-weighted spin-echo echo-planar-imaging sequence (DWI, TE_eff_/TR = 20 ms/3400 ms, slice thickness = 0.7 mm, number of slices = 17, 30 diffusion sensitized directions with a b value of 1500 s/mm^2^, δ = 3ms, Δ = 9ms, partial Fourier encoding with an acceleration factor of 1.51, field of view 12x15 mm^2^, matrix 96x128, in-plane resolution = 125 μm x 117 μm, FA = 90°-180° (spin-echo EPI), one saturation slice (sagittal), acquisition time = 7min 56s) and multi-slice multi-Spin-Echo (MSME) 2D T2-relaxometry (TE between 8 ms and 136 ms in increments of 8 ms, TR = 3100 ms, number of slices = 17, slice thickness = 0.7 mm, field of view 20x20 mm^2^, matrix = 172x172, FA = 90°-180° (spin-echo), acquisition time = 8min 53s) were performed.

### Image analysis

Image co-registration, region of interest (ROI) placement and microhemorrhage lesion segmentation were performed in the post-processing environment of Amira 5.4 (FEI Visualization Sciences Group, Burlington Massachusetts, USA). At first, the individual brains were manually segmented from the 3D GRE-weighted image datasets and converted into 8-bit image files. One individual mouse brain served as the registration mask onto which each of the 3D GRE-weighted image datasets of the other animals was registered. Registration was performed with the affine registration module of Amira using 7 degrees-of-freedom. The transform matrix for each registered data set was applied to the corresponding segmented data sets, per animal, and also to the lesion masks after manually segmenting microhemorrhages on all slices. This procedure enabled a final representation of microhemorrhages from all animals in a standard 3D space of this study.

Manual segmentation of microhemorrhages was performed on the T2* weighted datasets by comparing the two scans after reoxygenation (immediately after hypoxia and after 24 hours of reoxygenation) with the baseline scan before hypoxia showing any evidence of microhemorrhagic brain injury or otherwise conspicuous structural alterations from normal (184 coronal slice reformations and a total of 552 images per animal). A microhemorrhage was defined as a newly visible 3D GRE T2* hypointense lesion on either of the follow-up scans (immediately after hypoxia and after 24 hours of reoxygenation) when compared to the baseline scan 3D before induction of hypoxia. The strong structural contrast of the T2* weighted sequence allowed segmentation of the following neuroanatomical regions in standard space and in accordance with the MRM NeAt [17]: 1) olfactory bulb (OB), 2) basal ganglia (BG), 3) white matter (WM), 4) cortex and 5) brain stem. The total microhemorrhage number and microhemorrhage lesion volume were analyzed within each specific region.

For quantification of the T2 relaxation time MSME data were fitted after phase correction on a voxel-by-voxel basis with the monoexponential function A·e ^-(TE/T2)^ using a nonlinear least-squares fit procedure (MATLAB Release 2012b, The MathWorks, Inc., Natick, Massachusetts, United States). ADC and FA values were calculated with the postprocessing algorithm provided by Paravision 6.0 (Bruker Biospin GbmH, Ettlingen, Germany).

To evaluate potential region specific edema, ROIs in ADC, FA and T2 maps were analyzed within each of already mentioned anatomical regions.

### Histology

For histological analyses, anaesthetized animals were transcardially perfused with PBS (2 ml/min) for 5 min. Brains were removed, embedded into Tissue-Tek (Sakura Finetek, Staufen, Germany), and stored at -80°C until investigated. Coronal brain tissue sections (10 μm in thickness) were prepared using cryotomy. Tissue sections were fixed with zinc-based fixative containing 100 mM Tris (pH 7.4), 3 mM Ca(C_2_H_3_O_2_)_2_, 25 mM Zn(C_2_H_3_O_2_)_2_ and 35 mM ZnCl_2_ for 30 min and permeabilized with 0.5% saponin in PBS for 15 min. Brain slices were then incubated for 30 min in blocking buffer consisting of 10% goat serum (Dianova, Hamburg, Germany) in PBST (0.1% Tween-20 in PBS). Subsequently, slices were incubated overnight at 4°C with primary antibodies against hemoglobin (host species: rabbit; Biogenesis, Poole, UK), platelet endothelial cell adhesion molecule-1 (host species: rat; Pecam-1; BD, Heidelberg, Germany) or ZO-1 (host species: rabbit; Life Technologies, Darmstadt, Germany) followed by incubation with compatible Cy2- or Cy3-conjugated secondary antibodies (Dianova) for 1 hour. Alternatively, slices were incubated with Cy3-labeled goat anti-mouse IgG (Dianova) for 1 hour. All antibodies were diluted in LowCross-Buffer (Candor, Wangen, Germany). Slices were incubated for 10 min with 0.02% DAPI (Life Technologies) in PBS to stain nuclei. Stained sections on glass slides were then embedded in Mowiol mounting medium (Sigma-Aldrich, Steinheim, Germany). Fluorescence staining was recorded using an Olympus BX50 microscope with a Leica DC 500 camera.

### Statistical analysis

Paired Student's t-test analyses were performed to compare microhemorrhage lesion number/volume, T2 and ADC values between different time points. To test for an effect of interaction between neuroanatomical location and time point (on microhemorrhage load) a 3-factorial (OB, BG, WM) repeated measures (hypoxia vs. reoxygenation) ANOVA was performed. A two-sided alpha-level of 5% for statistical significance was applied. All data are expressed as the mean ± SD.

## Results

### Post-hypoxic reoxygenation dramatically increases the number and size of cerebral microhemorrhages

**Fig 1:**
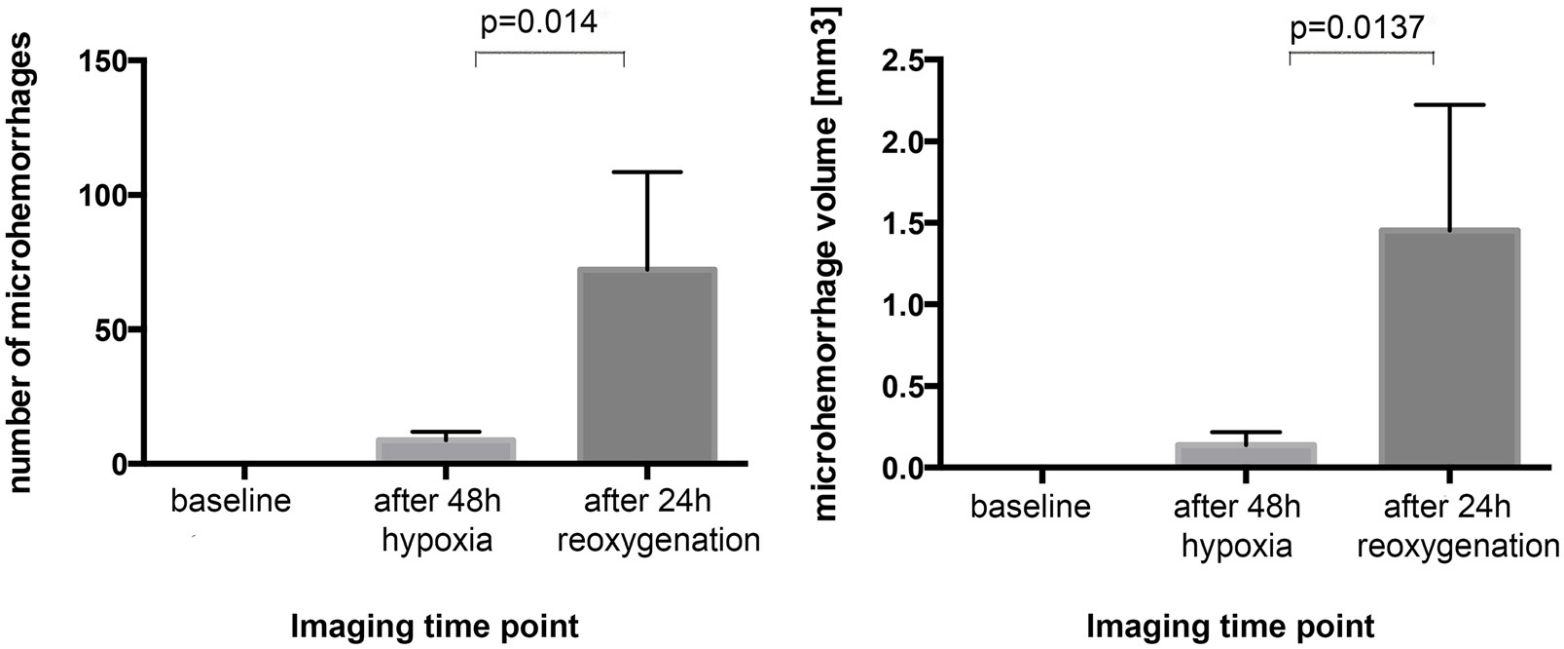
Post-hypoxic reoxygenation enhances total number and volume of brain microhemorrhages. Mice (*n* = 5) were exposed to 48 h of normobaric hypoxia (8% oxygen) followed by rapid reoxygenation and exposure to normoxic conditions (~21% oxygen) for 24 h. High-resolution MRI at 9.4T was performed to detect brain microhemorrhages before exposure to hypoxia, immediately after hypoxia and 24 h after onset of reoxygenation. Image analysis determined number and volume of microhemorrhages for each time point. At baseline no microhemorrhages are evident. Immediately after 48 h of hypoxia only few microhemorrhages have occurred whereas the substantial increase occurs 24 h after onset of reoxygenation.

Immediately after 48 hours of exposure to 8% oxygen (hypoxia) 8.8±3.1 brain microhemorrhages with a total volume of 0.14±0.08 mm^3^ per animal were detected. However, 24 hours after onset of reoxygenation, microhemorrhages largely and significantly increased in number (72±36; *p* = 0.014) and total volume (1.45±0.77 mm^3^; *p* = 0.014) (Fig 1).

### Microhemorrhages occur with a highly specific neuroanatomical distribution

**Fig 2:**
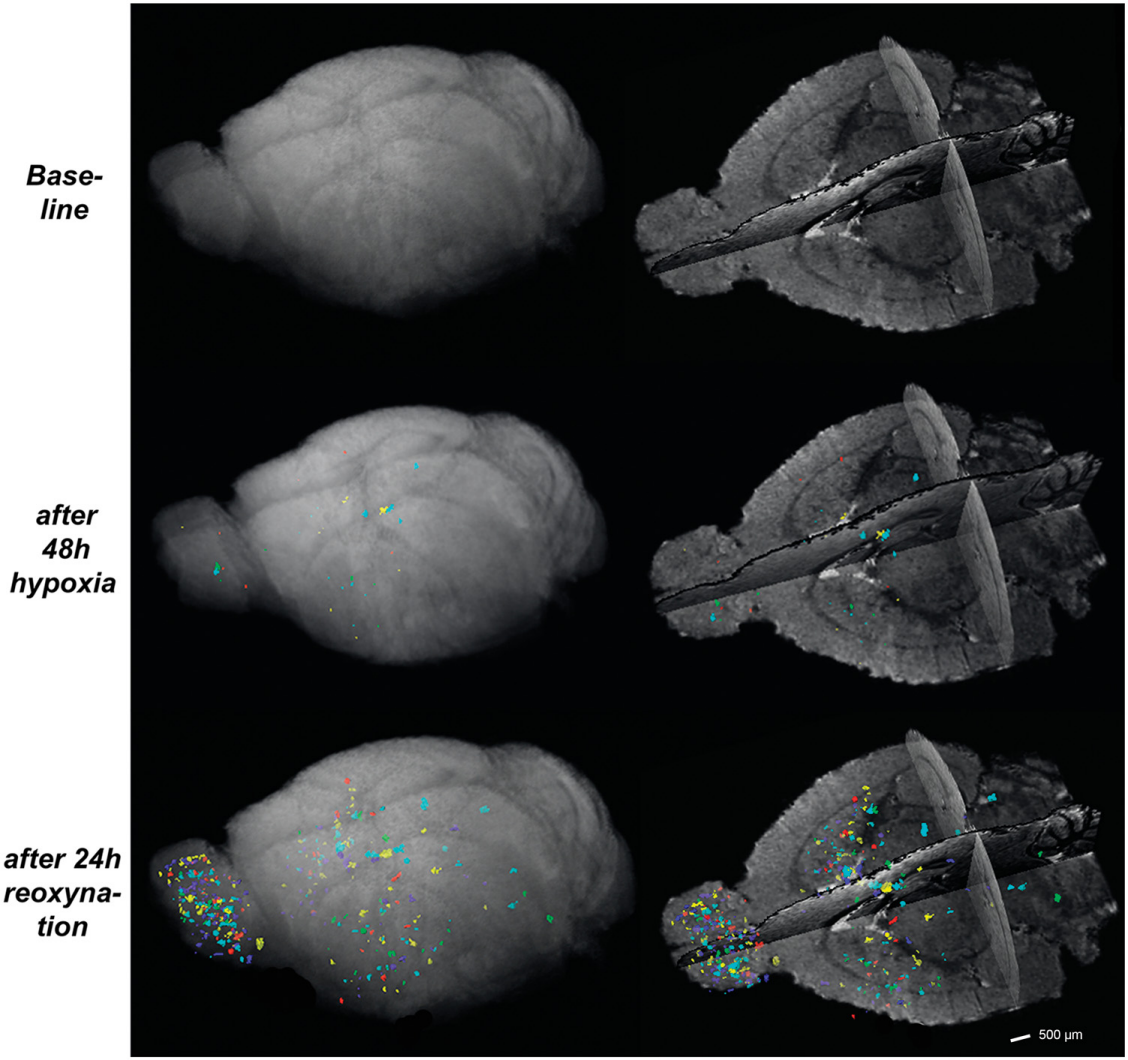
Spatial distribution of cerebral microhemorrhages. Mice (*n* = 5) were exposed to 48 h of normobaric hypoxia (8% oxygen) followed by rapid reoxygenation and exposure to normoxic conditions (~21% oxygen) for 24 h. High-resolution MRI at 9.4T was performed to detect brain microhemorrhages before exposure to hypoxia, immediately after hypoxia and 24 h after onset of reoxygenation. Overlay representation of microhemorrhages in a 3D standard space is shown for each time point. Each color represents one individual mouse. Anatomical clustering was observed within the olfactory bulb, the basal ganglia and cerebral white matter including the corpus callosum. A strong rostral predominance is observed in the olfactory bulb showing the highest lesion load. Scale bar = 500 μm.

Microhemorrhages did not appear equally distributed throughout the brain, but showed a strong spatial clustering. Lesions were mainly clustered in three distinct neuroanatomical regions: 1) the olfactory bulb (OB), 2) the basal ganglia (BG) and 3) the cerebral white matter including the corpus callosum (WM/CC) (Fig 2). Immediately after hypoxic exposure 2±1 lesions with a mean volume of 0.004±0.002 mm^3^ were found in OB, 4±2.5 lesions (volume 0.003±0.002 mm^3^) in BG, and 2±2 lesions (volume 0.007±0.006 mm^3^) in WM, while in other brain regions microhemorrhages were only rarely detected immediately after reoxygenation (cortex: 0.4±0.5 lesions (volume 0.0002±0.0002 mm^3^); brain stem: 0.4±0.9 lesions (volume 0.0005±0.0001 mm^3^)).

### Rostral predominance of reoxygenation-induced appearance of microhemorrhages

The number of microhemorrhages dramatically increased after 24 hours of reoxygenation in a region-specific manner with a strong predominance in the olfactory bulb (Fig 2).

**Fig 3:**
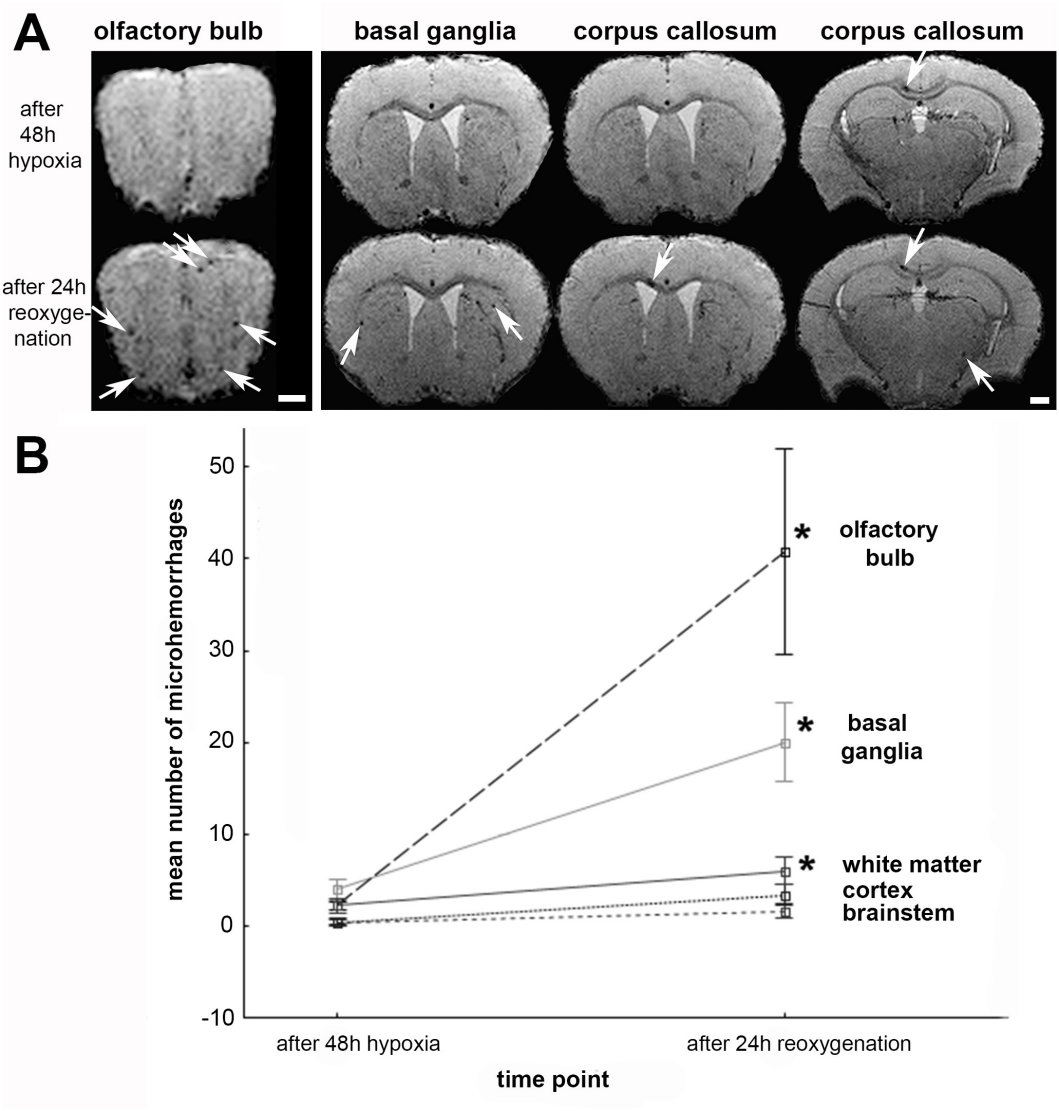
Temporal development of microhemorrhages in the CNS. Mice (*n* = 5) were exposed to 48 h of normobaric hypoxia (8% oxygen) followed by rapid reoxygenation and exposure to normoxic conditions (~21% oxygen) for 24 h. High-resolution MRI at 9.4T was performed to detect brain microhemorrhages before exposure to hypoxia, immediately after hypoxia and 24 h after onset of reoxygenation. (A) Immediately after 48 h of hypoxia (A, first row) very few microhemorrhages (white arrows) were present but increased significantly after 24 h of reoxygenation (**A**, second row), most notably in the olfactory bulb, and to a lesser extent in the basal ganglia and white matter, including the corpus callosum. Scale bar = 500 μm. **(B)** A significant increase of microhemorrhage number is marked with an asterisk (p<0.05). Please note the decreased venous contrast 48h after reoxygenation, which is caused by increased venous oxygen in the first minutes of reoxygenation. 24h after reoxygenation the venous contrast has normalized. Baseline images as well as images 48h after hypoxia and 24h after reoxygenation are displayed in S1 Movie. All mice displayed similar changes in T2* contrast.

While the lesion number increased 20-fold in the OB (40±25), the number of hemorrhages was only 5 times larger in the BG (20±9), and 3 times larger in WM/CC (6±3) respectively (Fig 3A). Accordingly, the total microhemorrhage lesion volume increased after reoxygenation by 20-fold in the OB (0.07±0.04 mm^3^), 11-fold in the BG (0.03±0.02 mm^3^), and 5-fold in WM/CC (0.03±0.02 mm^3^). In the cortex and brain stem, where only a small fraction of microhemorrhages was detected, the small differences concerning lesion number and size between the time point immediately upon hypoxic exposure and at 24 hours after reoxygenation were not significant.

The interaction between the factors of *TIME* (2 levels: immediately after reoxygenation vs. 24 hours after reoxygenation) and *LOCATION* (3 levels: OB, BG, WM) was significant (f-value = 8.52, *p* = 0.0003), indicating that the temporal progression of microhemorrhagic brain injury is strongly site specific. It can be readily appreciated in the 3D group plots (Fig 2) of total microhemorrhage load in standard space that the strongest site specific increase occurred in the OB (Fig 3B).

This qualitative visual finding is also statistically confirmed by comparing the temporal differences in lesion load between groups, comparing the number of microhemorrhages in the OB with the number of microhemorrhages in the BG and the WM/CC: The largest mean increase of 40±11 microhemorrhages in the OB was significantly higher compared to the increase in number of microhemorrhages in the WM/CC (6±1, *p* = 0.03) and showed a non-significant positive trend, when compared to the number of microhemorrhages in the BG (20±4 *p* = 0.06).

Microhemorrhages in the OB were mainly found in the outer regions, not in the granule layer. In the BG microhemorrhages are located in the grey matter along the internal and external capsule, and the cerebral peduncle. Microhemorrhages in the WM/CC are found in immediate vicinity of the lateral ventricles or in the white matter lying just beneath the cortex (Fig 3A).

Overall, these results indicate that full cerebrovascular injury in mice is not achieved immediately after hypoxic exposure but evolves over time upon tissue reoxygenation. Moreover, the OB and to a lesser extent also BG and WM/CC reveal a higher susceptibility to the formation of microhemorrhages in comparison to other brain sub regions.

### No evidence of regional or global brain edema on T2 or ADC/FA maps

No significant changes of T2, ADC or FA values were detected in any anatomical regions (OB, BG, WM, cortex, brainstem), which showed microhemorrhagic lesions, either immediately after 48 hours of hypoxia or after a further 24 hours of reoxygenation (Table 1).

**Table 1:** T2, ADC and FA values in the different anatomical regions are displayed.

T2 [ms] / ADC [x10^-3^ mm^2^/s] / FA	olfactory bulb	basal ganglia	white matter	cortex	brainstem
normoxia	42.2±0.3 / 0.6±0.03 / 0.24±0.004	39.2±0.5 / 0.53±0.03 / 0.19±0.01	37.3±0.5 / 0.51±0.02 / 0.55±0.02	39.6±1.1 / 0.56±0.03 / 0.14±0.01	36.4±0.9 / 0.52±0.03 / 0.22±0.003
24h after hypoxia	41.5±1.5 / 0.62±0.06 / 0.23±0.01	38.8±0.4 / 0.53±0.04 / 0.20±0.01	37±0.5 / 0.48±0.01 / 0.53±0.04	39±0.7 / 0.56±0.04 / 0.15±0.01	37.3±0.6 / 0.53±0.03 / 0.22±0.004
24 hour after reoxygenation	42.8±0.7 / 0.58±0.04 / 0.24±0.01	39.6±0.5 / 0.51±0.04 / 0.20±0.004	37.2±0.6 / 0.48±0.4 / 0.54±0.04	39.9±0.7 / 0.53±0.04 / 0.15±0.01	37.4±0.7 / 0.5±0.05 / 0.23±0.01

### Cerebral microhemorrhages upon hypoxia-reoxygenation are characterized by deposition of blood-borne molecules

**Fig 4:**
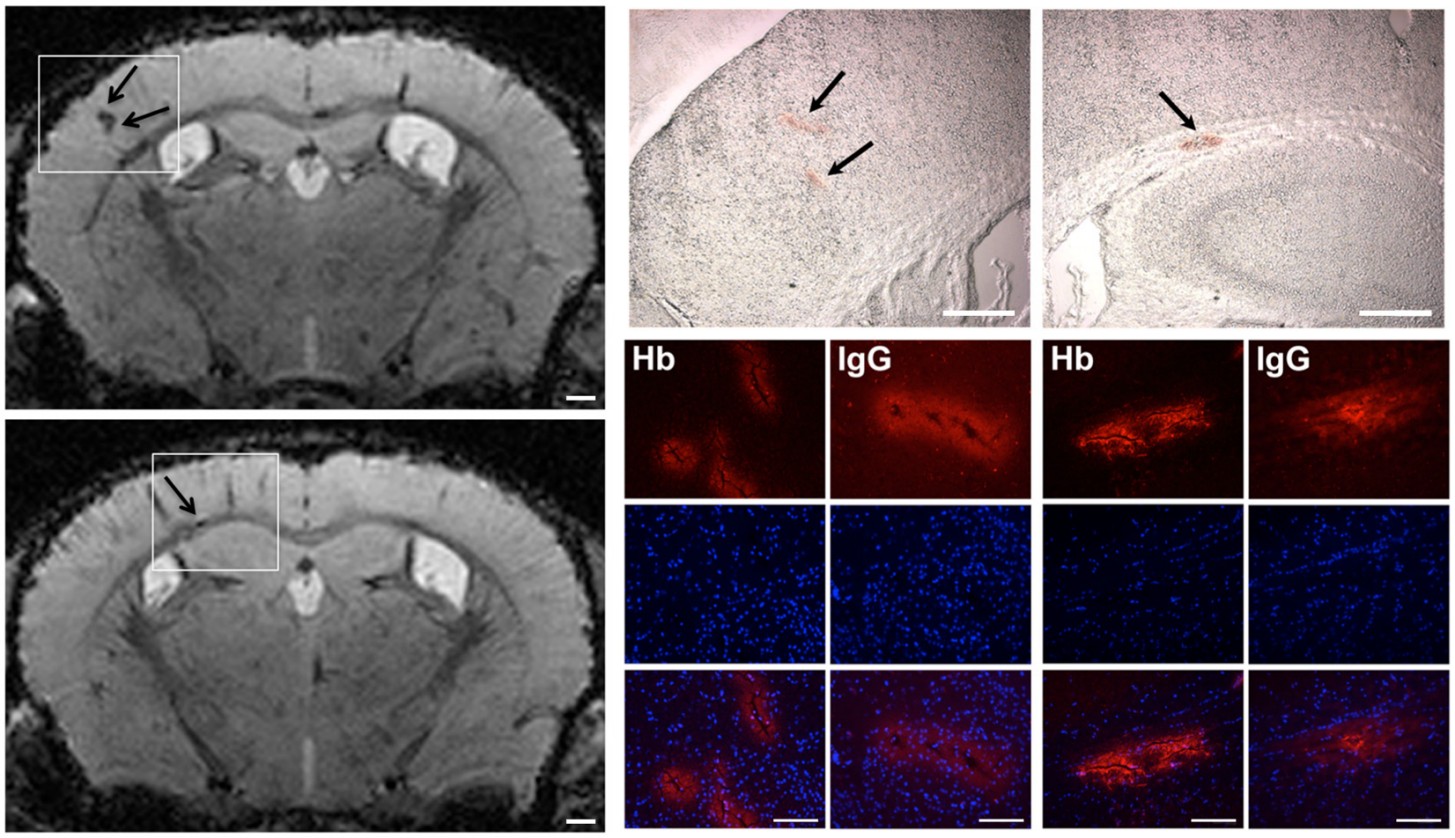
Histological detection of microhemorrhages in murine brain tissue upon hypoxia-reoxygenation stress. Mice (*n* = 5) were exposed to normobaric hypoxia at 8% oxygen for 48 h followed by 24 h reoxygenation phase. Subsequently, brains were removed, coronal cryosections were prepared, and histologically analyzed. Brightfield microscopy was used to validate mircohemorrhages seen on T2* images (scale bar = 500 μm) in unstained brain tissue sections (scale bar = 500 μm). Immunofluorescent staining of endogenous hemoglobin (Hb) and immunoglobulin G (IgG) was detected with fluorescence microscopy (scale bar = 50 μm). Cell nuclei were counterstained with DAPI (blue). Representative micrographs for one out of five analyzed mice are shown. Sections used for histological analyses correspond to Bregma -1.20 mm with reference to Franklin and Paxinos [18].

**Fig 5:**
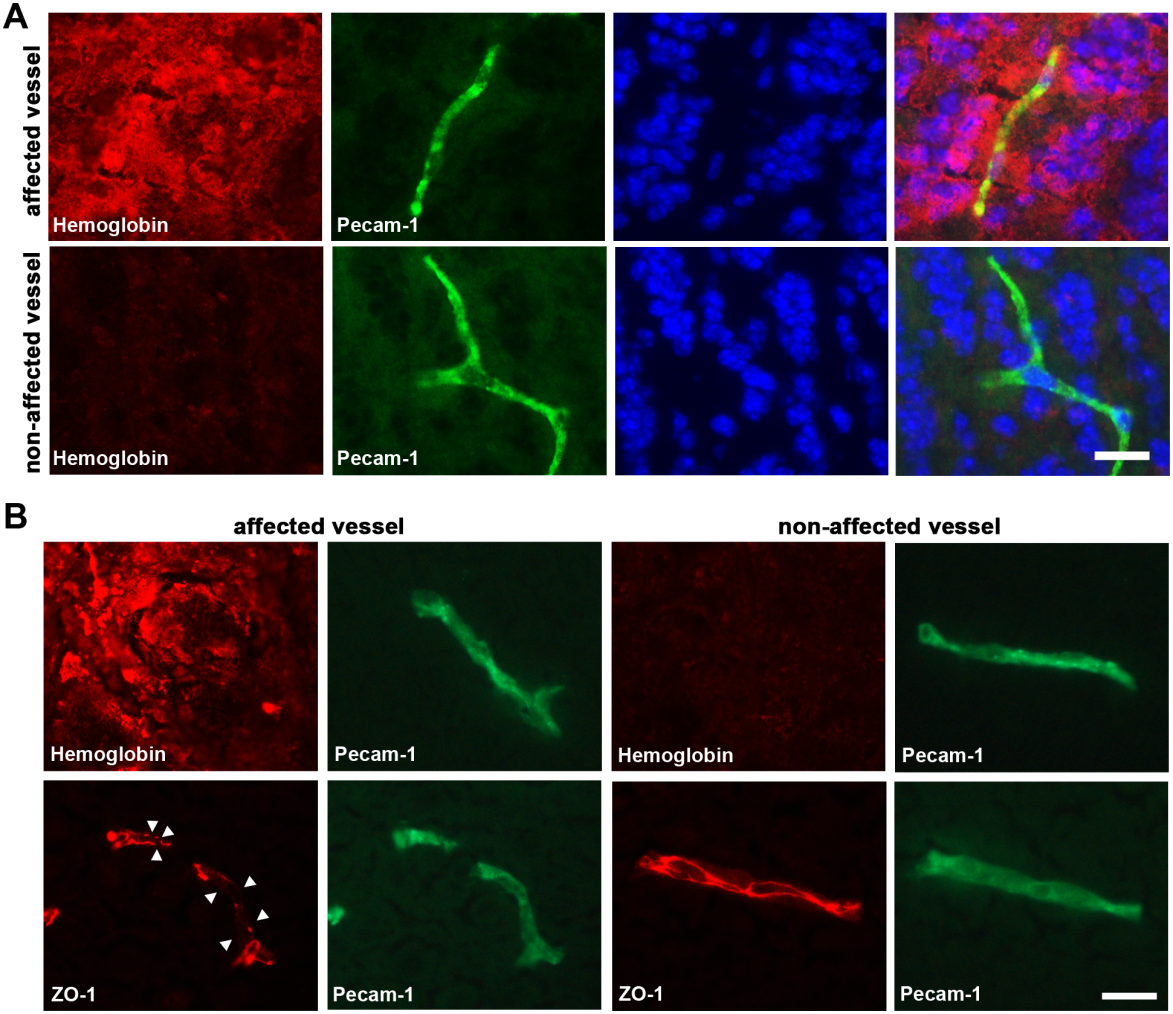
Microhemorrhages are associated with perivascular hemoglobin accumulation and rearrangement of TJ protein ZO-1. Mice (*n* = 5) were exposed to normobaric hypoxia at 8% oxygen for 48 h followed by 24 h reoxygenation phase. Brains were then removed, coronal cryosections were prepared, and immunfluorescent staining was performed. **(A)** Co-Immunofluorescent detection of hemoglobin and Pecam-1. Cell nuclei were counterstained with DAPI (blue). **(B)** Immunofluorescent detection of hemoglobin and ZO-1was performed on two separate, but adjacent serial tissue sections due to antibody incompatibility. Both sections were co-labeled for Pecam-1. Disruptions of the regular ZO-1 localization pattern are denoted by white arrowheads. Representative micrographs for one out of five analyzed mice are shown. Sections used for immunofluorescent analyses correspond to Bregma +3.90 mm. Scale bar = 20 μm.

To further characterize the underlying mechanisms causing cerebral microhemorrhages histological analyses were performed, and clearly indicated the presence of microhemorrhages in cerebral tissue of mice exposed to global hypoxia-reoxygenation (Fig 4). The anatomical location of microhemorrhages strongly overlapped with those determined by MRI (Fig 4). Microhemorrhage lesions were immunoreactive to endogenous hemoglobin and immunoglobulin G (Fig 4). Moreover, co-Immunofluorescence staining revealed that hemoglobin deposits were exclusively localized around cerebral microvessels (Fig 5A), indicating that microhemorrhage is due to microvascular damage.

### Microvessels with hemorrhagic lesions exhibit disrupted interendothelial tight junctions

To further analyze the vascular damage, localization and distribution of tight junction (TJ) proteins was characterized by immunofluorescence. The regular, continuous and sharp localization of the TJ protein ZO-1 along the interendothelial cell junctions was disrupted in blood vessels showing perivascular hemoglobin accumulation, suggesting that paracellular permeability of the BBB is enhanced during hypoxia-reoxygenation (Fig 5B).

In summary, BBB disruption was observed in areas of microhemorrhage occurrence without evidence of significant parenchymal brain edema at predilection sites.

## Discussion

In 1983 Dickinson et al. demonstrated the presence of microhemorrhages in postmortem brain specimens of HACE victims [19]. Recently, two studies using MRI techniques verified the occurrence of multiple microhemorrhages in brains of patients who had suffered from HACE, while failed to detect hemosiderin depositions in brains of AMS patients (2, 11). They postulated that cerebral microhemorrhages might serve as a novel diagnostic MRI sign for HACE even many months or years after the event (2, 11).

Unfortunately, temporal and spatial development as well as potential underlying mechanisms leading to high altitude brain edema and microhemorrhages are poorly understood, not least because appropriate animal models are only rarely available. Here, we present a mouse model in which we could recapitulate the occurrence of cerebral microhemorrhages after hypoxic exposure, *in vivo*, by MRI at 9.4T, and further verified their structural basis by conventional histological and immunofluorescent staining techniques. Our results might indicate that these vascular lesions are linked to processes during both hypoxia and reoxygenation as their number and size strongly increased during post-hypoxic reoxygenation. Moreover, we demonstrated that cerebral microhemorrhages upon hypoxia/reoxygenation stress occur at specific predilection sites with a remarkable predominance for the olfactory bulb, which has not been reported before.

### Potential mechanisms underlying the formation of brain microhemorrhages during HACE pathogenesis

In human HACE, it is difficult to acquire data about the temporal development of cerebral microhemorrhages or structural pathologies preceding microhemorrhages, as it is, for example, not feasible to perform MRI examinations at high altitude. In humans cerebral microhemorrhages are considered as ‘footprint’ of HACE and indicative of vascular damage that is only present in individuals who suffered from severe HACE, but not in AMS patients [2, 15]. Hypoxic conditions at high altitude are likely to increase levels of biochemical mediators of BBB permeability (e.g. VEGF, ROS) and to cause hemodynamic changes that might result in the loss of BBB integrity [5, 7–10]. However, the relative contribution of hypoxia-induced hemodynamic changes, mediators of vascular permeability such as VEGF, or ROS to microvascular damage is still elusive.

Our animal study now reveals the exact temporal appearance of microhemorrhages *in vivo*. Moreover, our histological analyses indicate that microhemorrhage development is closely associated with disruption of interendothelial TJs. While immediately after 48 hours of hypoxia only few cerebral microhemorrhages had occurred, their number and size strongly increased if mice were subjected to rapid and complete post-hypoxic reoxygenation. This finding could be of great importance for HACE therapy as the guidelines for prevention and treatment of AMS, HACE and HAPE released by the Wilderness Medical Society in 2014 strongly recommend the administration of oxygen in severe cases of AMS and HACE when descent is not feasible or delayed [20]. The acute delivery of supplemental oxygen as well as swift emergency helicopter evacuation to low altitude would undoubtedly lead to rapid tissue reoxygenation which is comparable to our experimental animal model. The observation of microhemorrhagic injury during reoxygenation suggests that reoxygenation-related mechanisms such as generation of ROS could enhance hypoxia-induced microvascular structural injury. Supporting evidence comes from experimental studies showing that hypoxia-reoxygenation strongly increased the production of injurious ROS by (micro)vascular endothelial cells *in vitro* [21–24] and cerebral endothelium *in vivo* [25] Furthermore, enhanced ROS formation was accompanied with TJ protein disruption and increased BBB permeability [26–29]. Similarly, Barthelmes et al. showed that a very large amount of human climbers developed retinal hemorrhages during ascent to Mt. Muztagh Ata (7546 m), whereas number and area of retinal hemorrhages were highest after return to base camp (4497 m) from a high altitude [30]. However, it is worth to mention that temporal profile and degree of reoxygenation (7100 m to 110 m within seconds in mice versus 7546 m to 4497 m within 12 h in human mountaineers) are not fully comparable. From their findings the authors hypothesized that prolonged exposure to hypoxic conditions at high altitude damage the vascular endothelium, which ultimately causes vessel rupture and leakage of blood in a delayed manner (36). However, it cannot be excluded that tissue hypoxia and reoxygenation synergistically enhance cerebrovascular injury through equal or distinct mechanisms.

### Spatial distribution of microhemorrhages

It is difficult to explain why the olfactory bulb is highly susceptible for microhemorrhagic brain injury after hypoxia/reoxygenation in mice. Up to now the effect of hypoxia to the olfactory bulb in mammals has hardly been studied and so far it has only been analyzed in very few neonatal hypoxia studies [31–33]. The olfactory bulb is a central part of the rodent brain, as olfaction is a key sense in mammals, homing over 1000 genes for the detection of odors out of approximately 30000 genes [34]. Additionally, approximately 5% of the whole brain weight is covered by the olfactory bulb [35]. The latter has also been studied extensively in electrophysiology, but has mostly been ignored in disease models. In mammals the olfactory bulb plays a central role as odor is indispensable to daily life. During brain evolution the central function of the olfactory bulb in mammals has shifted in humans towards other brain regions. Therefore, several areas in the human brain have been proposed to represent the mammalian olfactory bulb [36–38]. The human retinal tissue for example has been compared to the mammalian olfactory bulb because of its similar intrinsic circuitry [37], and has also been demonstrated to be highly susceptible to hypoxia/reoxygenation-induced formation of hemorrhages [30].

In addition to the olfactory bulb, post-hypoxic microhemorrhages occur in the white matter, including the corpus callosum, and the basal ganglia, also with a strong increase following rapid reoxygenation.

In human HACE survivors, microhemorrhages show a high predilection for the splenium of the corpus callosum [2, 15]. In our animal model microhemorrhages were also evident in the corpus callosum. A possible explanation for this predilection site might be rooted in its vascular supply: small, short, perforating arteries without adrenergic tone, which might make them more susceptible to hypoxia-induced vasodilatation, autoregulatory failure, and thus hyperperfusion [39, 40].

In severely affected patients microhemorrhages were not only seen in the corpus callosum, but also in the periventricular white matter [15], which can be considered as watershed areas of cerebral perfusion. In the mouse, watershed regions are located, for example, along the external capsule in the basal ganglia [41].

Altogether, cerebral microhemorrhages in our animal model occurred in similar areas of predilection compared to humans, such as the corpus callosum and adjacent white matter. However, the strongest vulnerability was seen in the OB marking one significant difference between mice and humans.

### Is the cerebral edema of global or rather focal nature?

Microhemorrhages showed a multifocal pattern and disrupted TJs, as a sign of BBB impairment, were clearly evident in sites of microhemorrhage. Interestingly, global and regional quantitative T2 and ADC/FA values, even in regions with strongest microhemorrhagic injury such as in the OB, were not significantly different from baseline. These findings strongly suggest that neither cytotoxic nor vasogenic brain edema were present immediately after 48 hours hypoxia or 24 hours upon onset of reoxygenation. However, we cannot exclude, that cerebral edema due to hypoxic exposure is rapidly reversible or possibly masked by T2/T2* signal reduction induced by extravascular hemoglobin deposits or by partial volume effect.

This experimental observation is in line with the few studies in humans not reporting AMS related T2/ADC alterations in white and grey matter regions [13, 14], but also contrasts other human studies reporting minor increases/decreases in T2/ADC within the splenium of the corpus callosum [1, 11] or increases of ADC in the splenium of the corpus callosum in AMS subjects [12]. Hunt et al. additionally showed decreases in ADC in grey matter, basal ganglia and white matter in AMS subjects [12].

Our experimental observations may be helpful to address this discrepancy between human studies. Structural injury of the BBB is an undisputed requirement for (micro)hemorrhagic brain injury after hypoxia and was verified histologically in our study by consistently observing TJ disruption. The spatial distribution of BBB disruption and consequent occurrence of microhemorrhages, however, did not occur homogeneously over affected brain regions but were scattered multifocally. Furthermore, other investigators have been documented by the use of low molecular weight permeability tracer (e.g. sodium fluorescein) that BBB hyperpermeability *in vivo* emerges neither globally nor regionally, but occurred in small scattered multiple foci [42]. These experimental observations by us and others and together with the discrepancy in the human data indicate that cerebral edema in HACE is difficult to detect in a robust manner suggesting that BBB impairment might occur multifocally. It is probably transient and finally masked from reliable *in vivo* MRI detection by T2/T2* signal decrease induced by extravascular hemoglobin deposits.

## Conclusions

In conclusion, we present an animal model of hypoxia/reoxygenation that partially reflects the conditions leading to HACE in humans. We could demonstrate that systemic inspiratory hypoxia caused brain microhemorrhages, whose number substantially increased upon post-hypoxic reoxygenation. Cerebral microhemorrhages, described as structural footprint of HACE, have been located in similar predilection sites compared to humans without evidence of a global parenchymal edema, pointing to a multifocal rather than a global pathological mechanism of HACE. Additionally, we draw attention to the olfactory bulb, a neglected region in the rodent brain, which is highly susceptible to hypoxia/reoxygenation and encourage further studies investigating this anatomical region in animal models of high altitude sickness.

## Supporting Information

S1 Movie Representative T2* datasets of one representative mouse 3 days prior to hypoxic exposure, 48h after hypoxia and 24h after reoxygenation are displayed. Note the drastic increase of microhemorrhages 24h after reoxygenation compared to 48h after hypoxia.
(MP4)
